# Re-Understanding the Mechanisms of Action of the Anti-Mycobacterial Drug Bedaquiline

**DOI:** 10.3390/antibiotics8040261

**Published:** 2019-12-11

**Authors:** Jickky Palmae Sarathy, Gerhard Gruber, Thomas Dick

**Affiliations:** 1 Department of Medicine, Yong Loo Lin School of Medicine, National University of Singapore, Singapore 117597, Singapore; e0149077@u.nus.edu; 2 School of Biological Sciences, Nanyang Technological University, Singapore 637551, Singapore; GGrueber@ntu.edu.sg; 3 Center for Drug Discovery and Innovation, Hackensack Meridian Health, Nutley, NJ 07110, USA; 4 Department of Medical Sciences, Hackensack Meridian School of Medicine at Seton Hall University, Nutley, NJ 07110, USA; 5 Department of Microbiology and Immunology, Yong Loo Lin School of Medicine, National University of Singapore, Singapore 117545, Singapore

**Keywords:** tuberculosis, Bedaquiline, diarylquinoline, TBAJ-876, F-ATP synthase, *c*-subunit, ε-subunit, uncoupler

## Abstract

Bedaquiline (BDQ) inhibits ATP generation in *Mycobacterium tuberculosis* by interfering with the F-ATP synthase activity. Two mechanisms of action of BDQ are broadly accepted. A direct mechanism involves BDQ binding to the enzyme’s *c*-ring to block its rotation, thus inhibiting ATP synthesis in the enzyme’s catalytic α_3_β_3_-headpiece. An indirect mechanism involves BDQ uncoupling electron transport in the electron transport chain from ATP synthesis at the F-ATP synthase. In a recently uncovered second direct mechanism, BDQ binds to the enzyme’s ε-subunit to disrupt its ability to link *c*-ring rotation to ATP synthesis at the α_3_β_3_-headpiece. However, this mechanism is controversial as the drug’s binding affinity for the isolated ε-subunit protein is moderate and spontaneous resistance mutants in the ε-subunit cannot be isolated. Recently, the new, structurally distinct BDQ analogue TBAJ-876 was utilized as a chemical probe to revisit BDQ’s mechanisms of action. In this review, we first summarize discoveries on BDQ’s mechanisms of action and then describe the new insights derived from the studies of TBAJ-876. The TBAJ-876 investigations confirm the *c*-ring as a target, while also supporting a functional role for targeting the ε-subunit. Surprisingly, the new findings suggest that the uncoupler mechanism does not play a key role in BDQ’s anti-mycobacterial activity.

## 1. Introduction 

Tuberculosis (TB), caused by *Mycobacterium tuberculosis* (Mtb) [1,2], is the leading infectious disease killer worldwide and thus a major global health concern [1,3]. One of the contributing factors to this ongoing health crisis is the alarming rise of drug resistance [3]. Unfortunately, the incidence of drug-resistant TB has been predicted to continue rising [3,4]. To combat drug-resistant TB, the anti-TB drug Bedaquiline (BDQ) was fast-tracked for approval by the US Food and Drug Administration (FDA) in 2012 [5,6]. This drug is conditionally administered for the treatment of multi-drug-resistant TB (MDR-TB) [6,7,8], which is defined as resistance against the first-line drugs isoniazid and rifampicin [9].

**Figure 1:**
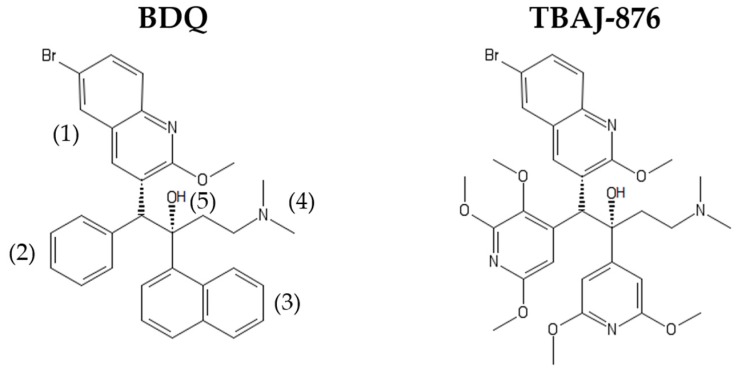
Structures of Bedaquiline (BDQ) and TBAJ-876. BDQ’s quinoline (1), dimethylamino (4), and hydroxyl (5) moieties are retained in TBAJ-876. However, the parental drug’s phenyl (2) and naphthalene (3) moieties are replaced by the 2,3,5-trialkoxypyridin-4-yl and 3,5-dialkoxypyridin-4-yl moieties, respectively.

BDQ (Figure 1) is a first-in-class diarylquinoline that is highly active against Mtb. It displays potent in vitro activity against both drug-sensitive and drug-resistant strains of Mtb (Minimum Inhibitory Concentration (MIC) of 0.002–0.013 µg/mL [10,11]). This potent activity holds true in vivo where BDQ has demonstrated accelerated sterilizing activity [12], with four months of BDQ treatment being as efficacious as six months of first-line drug treatment of Mtb-infected mice [13]. Importantly, BDQ also displays bactericidal activity against non-replicating Mtb at therapeutically attainable concentrations [14,15]. In the clinical setting, the usage of BDQ has produced promising results. Studies have shown that the usage of the drug for drug-resistant TB treatment improved sputum conversion and reduced chemotherapy duration and relapse [16,17,18,19]. Hence, new BDQ-containing drug regimens are currently being explored in Phase III clinical trials, such as the STREAM and SimpliciTB trials [20]. One such trial, the Nix-TB trial, resulted in the recent US FDA approval of the BDQ-pretomanid-linezolid six months, all-oral regimen for the treatment of drug-resistant TB [21,22].

BDQ carries out its anti-mycobacterial activity by inhibiting ATP synthesis, a process that is crucial to the growth of Mtb and to its survival in its non-growing dormant state [15,23,24]. Inhibition of ATP synthesis occurs through the drug’s interference with mycobacterial F-ATP synthase activity, which consequently leads to the depletion of bacterial ATP [25,26]. Interestingly, the bactericidal activity of BDQ is delayed, i.e., cell death does not occur immediately upon depletion of ATP [27]. Metabolomic, transcriptomic, and proteomic analyses of BDQ’s effect on Mtb have shed some light on the complexity of the intra-bacterial follow-on events after inhibition of ATP synthesis. A study by Koul et al. showed that BDQ inhibits protein and DNA synthesis in a dose-dependent manner, despite not directly targeting any component of these processes [27]. A recent study by Wang et al. showed that BDQ affects nitrogen metabolism, specifically the activity of glutamine synthetase as this enzyme is highly sensitive to changes in intra-bacterial ATP content [28]. These findings suggest that BDQ’s inhibition of ATP synthesis causes differential inhibition of various ATP-dependent metabolic processes. This ‘extended mechanism of action’ of BDQ is consistent with the emerging concept that the modulation of a primary target by an antibiotic triggers a series of intra-bacterial follow-on events resulting in the corruption of multiple cellular processes [29].

Although BDQ is a highly efficacious drug, it has pharmacological and toxicological liabilities. The drug is highly lipophilic (cLogP = 7.25), which can contribute to long terminal half-life due to its accumulation within tissues via phospholipidosis [30]. Another issue is the drug’s inhibition of cardiac hERG potassium ion channels which can potentially lead to QT interval prolongation [31]. This raises concerns regarding co-administration of BDQ with other QT interval-prolonging drugs (e.g., fluoroquinolones) [32]. To address these liabilities, a medicinal chemistry campaign was conducted [33,34,35,36,37]. This chemistry-driven work, using Mtb whole-cell activity as a read-out, resulted in the discovery of 3,5-dialkoxypyridine analogues of BDQ [37]. TBAJ-876 (Figure 1) was selected from this series to progress to pre-clinical development due to its lower lipophilicity, lower hERG ion channel inhibition, higher clearance and retention of BDQ’s potent activity against Mtb [37]. The reason for TBAJ-876′s lower lipophilicity is its different structure compared to BDQ. The parental drug’s naphthalene and phenyl moieties are replaced by the more hydrophilic 2,3,5-trialkoxypyridin-4-yl and 3,5-dialkoxypyridin-4-yl moieties, while only the quinoline, dimethylamino, and hydroxyl moieties are retained in the compound (Figure 1). Recent work on the mechanism of action of TBAJ-876 provided an opportunity to utilize the compound as a chemical probe to revisit BDQ’s mechanisms of action. In this review, we summarize and discuss findings on BDQ’s mechanisms of action before the discovery of TBAJ-876, and then describe the new insights into the mechanisms derived from studies of the new compound.

## 2. Mechanisms of Action of BDQ—Before TBAJ-876′s Discovery

ATP synthesis, the process targeted by BDQ, occurs through a complex metabolic pathway known as oxidative phosphorylation. This essential pathway starts off with activity along the electron transport chain (ETC) [38]. In the ETC, NADH or succinate donate electrons to membrane-associated protein complexes which subsequently pass these electrons to terminal oxidases or reductases through the electron-carrier menaquinone [38]. This activity is accompanied with the pumping of protons from the cytoplasm to the periplasm by some of the complexes of the ETC. The pumping of protons generates a transmembrane pH gradient and contributes to the membrane potential, both of which are components of the proton motive force [38]. This force drives the rotation of the F-ATP synthase *c*-ring, which consequently drives ATP synthesis at the enzyme’s α_3_β_3_ catalytic headpiece via the enzyme’s central stalk subunits γ and ε [38,39,40,41].

### 2.1. Stalling of Rotation of the Mycobacterial F-ATP Synthase c-Ring

BDQ is a direct inhibitor of the mycobacterial F-ATP synthase [10,25,26]. The drug’s first binding site on the enzyme was shown to be the *c*-subunit, nine copies of which make up the *c*-ring [42]. This binding site was initially uncovered through extensive spontaneous mutant generation studies, which showed that BDQ resistance mutations were located at the D28, E61, A63, and I66 amino acid residues of the Mtb *c*-subunit [10,43,44]. Binding and biochemical studies showed that *c*-subunit mutations prevented BDQ binding to the *c*-subunit and thus reduced the drug’s inhibitory effect on the F-ATP synthase’s activity, hence providing further evidence that the drug targets the *c*-ring as its mechanism of action [25]. A subsequent structural and computational study by Preiss et al. resolved the crystal structure of the *Mycobacterium phlei* F-ATP synthase *c*-ring in complex with BDQ [42]. This study showed that BDQ binds to a cleft between two *c*-subunits of the *c*-ring and that this binding site is composed of the *M. phlei* counterparts of the Mtb *c*-subunit D28, E61, A63, and I66 amino acid residues [42]. Furthermore, the study showed that multiple BDQ molecules can be bound to the *c*-ring simultaneously, depending on the drug’s concentration [42]. While bound to the *c*-ring, interplay between BDQ molecules can occur, as suggested by the recent finding that *c*-ring binding affinity of a BDQ molecule is increased when there is complementary binding of an additional BDQ molecule [45].

BDQ binding to the *c*-ring has been proposed to inhibit the mycobacterial F-ATP synthase’s ATP synthesis activity by stalling *c*-ring rotation. This is due to the complex of BDQ bound to a *c*-subunit being sterically unfavorable to pass through the interface between the enzyme’s *a*-subunit and *c*-ring [42]. Furthermore, BDQ’s interaction with the essential E61 amino acid residue blocks it from carrying out ion exchange which is required to facilitate proton flow down the transmembrane pH gradient [42].

The *c*-ring of the mycobacterial F-ATP synthase is a target of importance for the activity of BDQ. This is reflected in studies which show that high levels of BDQ resistance (10–100-fold MIC) are conferred by in vitro isolated spontaneous *c*-subunit missense mutations [43,44]. The recent emergence of *c*-subunit resistance mutations in clinical isolates from BDQ-treated TB patients further affirms this notion [46].

### 2.2. Uncoupling Electron Transport from ATP Synthesis

In addition to directly inhibiting the mycobacterial F-ATP synthase, BDQ functions as an uncoupler of electron transport from ATP synthesis at high drug concentrations [47,48]. Biochemical studies on *Escherichia coli* liposomes by Hards et al. showed that BDQ functions as an uncoupler by behaving as a H^+^/K^+^ anti-porter [48]. BDQ translocates protons in a similar manner as the lipophilic weak base ellipticine [48]. This allows the protonated form of BDQ to cross the hydrophobic core of the mycobacterial membrane. BDQ’s translocation of protons reduces the proton motive force by dissipating its transmembrane pH gradient component, thereby severing the link between ETC activity and ATP synthesis [47,48]. Interestingly, BDQ is unlike typical uncouplers as it requires localization to a protein target (F-ATP synthase) to exert its effect [47,48].

Importantly, the H^+^/K^+^ anti-porter mechanism, put forward by Hards et al., was able to account for the peculiar lack of effect on the membrane potential during BDQ treatment [47]. This could not be accounted for by previous proposals of BDQ’s uncoupler activity, such as BDQ behaving as a cationic protonophore [49] or causing uncontrolled proton leakage by perturbing the interface between the *a*-subunit and the *c*-ring [47]. Interestingly, the notion that BDQ is a H^+^/K^+^ anti-porter was also proposed by Nath [50], based on his theory that the flow of protons down the transmembrane pH gradient is accompanied by anion/counter-cation translocation [51]. Nath’s hypothesis also stated that BDQ’s H^+^/K^+^ anti-porter activity occurs from the vicinity of its binding site on the *c*-ring [50].

Previous work on uncouplers has shown that their effect on the proton motive force leads to the reduction of intra-bacterial ATP content and altered intra-bacterial pH, both of which affects bacterial viability [52,53]. Therefore, BDQ’s uncoupler activity was proposed to be crucial for the drug’s bactericidal activity against Mtb [47,48]. This is supported by the observation that the high concentrations needed for the uncoupler activity are within the range of concentrations at which the drug’s bactericidal activity occurs (≥30-fold MIC) [27,47]. Hence, the uncoupling of electron transport from ATP synthesis is viewed as a mechanism that is important for the mycobactericidal activity of BDQ.

### 2.3. Disrupting the Mycobacterial F-ATP Synthase ε-Subunit’s Function of Linking c-Ring Rotation to ATP Synthesis

Recent research by our groups have uncovered the ε-subunit as a second target of BDQ on the mycobacterial F-ATP synthase. BDQ’s targeting of the ε-subunit was first revealed through binding studies conducted by Biukovic’ et al. [54]. The authors showed that BDQ binds to the isolated Mtb ε-subunit at the region around the W16 amino acid residue [54]. A genetic approach was utilized in a subsequent study where two mutant *Mycobacterium smegmatis* strains were engineered, one overexpressing the ε-subunit and the other harboring a mutation of the ε-subunit W16 residue, and tested for their susceptibility to BDQ [55]. The findings showed susceptibility shifts induced by the engineered genetic alterations, suggesting activity of the drug on the ε-subunit in intact bacteria [55].

Recently, the solution Nuclear Magnetic Resonance (NMR) structure of the Mtb ε-subunit was resolved [56]. This structure, together with genetic and biochemical studies, showed that the Mtb ε-subunit plays a novel role in ATP synthesis by linking *c*-ring rotation to ATP synthesis at the α_3_β_3_-headpiece [56,57,58]. Critical to this function is the Mtb ε-subunit’s inter-domain amino acid interaction network, which transmits information on *c*-ring rotation throughout the subunit and to its C-terminus. The C-terminus can adopt an extended conformation to interact with the α_3_β_3_-headpiece to pass on this information. This process is proposed to contribute to the initiation of ATP synthesis at the α_3_β_3_-headpiece [56,58]. Through NMR titration, BDQ’s binding site on the Mtb ε-subunit was uncovered to be the A10–W16 amino acid region [56]. This study also showed that BDQ binding induces intra-protein structural changes that affect the subunit’s amino acid interaction network. By corrupting the intra-subunit communication network, BDQ binding appears to inhibit the ε-subunit’s ability to link *c*-ring rotation to ATP synthesis [56].

However, there are two experimental findings that put into question the contribution of targeting the ε-subunit to BDQ’s inhibition of F-ATP synthase activity. Firstly, BDQ has a dissociation constant (K_d_) of 19.1 μM with the isolated ε-subunit protein, suggesting only moderate binding affinity for the drug [54]. This is in contrast to the drug’s K_d_ of 500 nM for the *c*-subunit [25] and nM-potency in terms of IC_50_ for ATP synthesis activity [25] and MIC [10,11]. Secondly, spontaneous BDQ resistance mutations in Mtb map only to the *c*-subunit but not the ε-subunit [43,44]. Collectively, these two findings suggest that targeting the ε-subunit may play a secondary, perhaps even negligible role in the on-target activity of BDQ when compared to its *c*-ring targeting mechanism.

## 3. Insights into BDQ’s Mechanisms of Action Derived from TBAJ-876 Studies

TBAJ-876 was discovered through a chemistry-driven approach, using Mtb whole cell activity as readout. This novel BDQ analogue is structurally (Figure 1), as well as physico-chemically [37], distinct from the parental drug. Thus, TBAJ-876 provided an opportunity to revisit the mechanisms of action of BDQ. The new analogue was shown to be a potent biochemical inhibitor of mycobacterial F-ATP synthase activity and to retain BDQ’s anti-mycobacterial potency, including its bactericidal activity [59,60]. Based on these findings, questions could be raised on the compound’s retention of BDQ’s mechanisms of action. If targeting the ε-subunit is not required for effective inhibition of the mycobacterial F-ATP synthase, one would assume that this property should have been easily lost during empirical optimization. On the other hand, if BDQ’s uncoupler property is indeed a mechanism critical to BDQ’s activity, this property should have been retained during the chemical derivatization process.

### 3.1. TBAJ-876 Retained BDQ’s Targeting of Both the c-Ring and ε-Subunit

The biochemical study of the mechanisms of action of TBAJ-876 confirmed that the compound inhibits the mycobacterial F-ATP synthase. The findings showed a correlation between TBAJ-876′s more potent inhibition of the mycobacterial F-ATP synthase’s ATP synthesis activity and its lower MIC compared to BDQ, which suggested that the strength of mycobacterial F-ATP synthase inhibition influences the drug’s anti-mycobacterial activity [59]. The same study also confirmed that targeting the mycobacterial F-ATP synthase *c*-ring is critical to BDQ’s anti-tubercular activity. This was reflected by the finding that high levels of resistance against both TBAJ-876 and BDQ are conferred by *c*-subunit missense mutations isolated against TBAJ-876 [59].

Interestingly, TBAJ-876 was found to retain targeting of the mycobacterial F-ATP synthase ε-subunit. NMR titration studies showed that TBAJ-876 not only binds to the same binding region as BDQ but also induces similar structural changes inside the ε-subunit [59]. Furthermore, susceptibility testing of TBAJ-876 against engineered *M. smegmatis* strains, either over-expressing the ε-subunit or harboring an amino acid alteration at the ε-subunit W16 amino acid residue, showed similar MIC shifts observed for BDQ. This suggested that TBAJ-876 interacts with the ε-subunit not only in vitro but also in intact bacteria [59]. These findings were somewhat surprising as one may have expected that TBAJ-876 had lost BDQ’s—only moderate—affinity for the ε-subunit owing to its different structure: the compound lacks BDQ’s phenyl and naphthalene moieties (Figure 1) which are proposed to interact with the Mtb ε-subunit’s W16, S17, and F86 amino acid residues [56].

The finding that TBAJ-876 retained BDQ’s dual targeting of both the *c*-ring and ε-subunit of the mycobacterial F-ATP synthase supports an on-target mechanism of action model in which targeting both components of the enzyme is required for effective inhibition of enzyme activity. It is interesting to note that the F-ATP synthase has been previously shown to possess the ability to overcome inhibition of *c*-ring rotation by a small molecule. This was uncovered through studies on N, N’-Dicyclohexylcarbodiimide (DCCD), a known F-ATP synthase inhibitor that binds to a similar binding site as BDQ on the *c*-subunit. These studies showed that the *E. coli* F-ATP synthase is able to undergo transient rotations that allow for low enzyme activity when DCCD is bound to the *c*-ring [61]. While transient rotations of the mycobacterial F-ATP synthase have yet to be uncovered, BDQ’s inhibition of the ε-subunit may be required to nullify the contribution of possible transient rotations to the enzyme’s ATP synthesis.

The studies of BDQ and TBAJ-876 on engineered *M. smegmatis* ε-subunit mutants suggest that both antibiotics interact with the subunit in vivo at low concentrations. How can this observation be reconciled with the drug’s moderate affinity for the ε-subunit in vitro (K_d_ of 19.1 μM)? The measurement of the drug’s binding affinity to the subunit is based on the isolated recombinant ε-subunit protein [54], which is likely to differ in conformation from the dynamic forms of the subunit in the intact F-ATP synthase complex [56]. Thus, it is plausible that BDQ’s binding affinity for the ε-subunit may be higher for the subunit’s conformation(s) presented in situ, which would be consistent with the effects of the drug observed in intact bacteria possessing engineered mutations in the ε-subunit [55].

If targeting the ε-subunit is required for BDQ’s activity, why is it not possible to isolate BDQ resistance mutations in the ε-subunit? The mechanism of action and resistance against DCCD could provide an insight into this peculiar observation. Besides targeting the *c*-ring [62,63,64], DCCD also targets the β-subunit [65,66] of the F-ATP synthase. However, studies on *E. coli* [64] and *Streptococcus faecalis* [62] have shown that spontaneous DCCD resistance mutations can only be isolated in the *c*-subunit but not the β-subunit. Interestingly, a mutational study of DCCD’s binding site on the *E. coli* β-subunit (E181 residue) showed that site-directed mutagenesis of this key residue resulted in loss of the subunit’s activity [67]. This suggests that the bacterium cannot tolerate spontaneous resistance mutations in the β-subunit due to their detrimental effect on F-ATP synthase activity. This may also be the case for BDQ and the ε-subunit. Site-directed mutagenesis of the W16 amino acid in BDQ’s binding site on the ε-subunit has been shown to cause a strong reduction of ATP synthesis by the mycobacterial F-ATP synthase [56]. This effect of the amino acid alteration on enzymatic activity is consistent with the mutation having a destabilizing effect on the subunit’s amino acid interaction network, which is required for the ε-subunit’s ability to link *c*-ring rotation to ATP synthesis at the α_3_β_3_-headpiece [56]. This suggests that resistance-conferring missense mutations in the ε-subunit are not tolerated by the bacterium, which may explain the inability to isolate BDQ resistance mutations in this subunit.

### 3.2. TBAJ-876 Did Not Retain BDQ’s Uncoupler Activity 

BDQ’s uncoupler activity is proposed to contribute to the bactericidal activity of the drug [47,48]. Hence, TBAJ-876 should have retained this property since the compound was uncovered to possess similar bactericidal activity as BDQ [60]. However, TBAJ-876 was found to have lost BDQ’s uncoupler activity. TBAJ-876 was shown to have a much weaker effect than the parental drug in dissipating the transmembrane pH gradient in *M. smegmatis* inverted membrane vesicles [60]. Further analyses showed that the compound was less effective than BDQ in crossing the mycobacterial membrane [60]. This could be due to the compound’s lower lipophilicity compared to the parental drug since high lipophilicity is required by protonophores for moving through lipid-rich membranes to translocate protons [68]. Collectively, these findings suggest that the uncoupler activity of BDQ is not required by the drug to exert bactericidal activity. This notion regarding the role of BDQ’s uncoupler activity is further supported by the drug’s inability to induce carbonyl cyanide m-chlorophenyl hydrazine (CCCP)-like mycobacterial killing during the initial phase of BDQ treatment [27,53], despite the drug possessing similar protonophore activity as the bona fide uncoupler [47,48].

## 4. Conclusions

**Figure 2:**
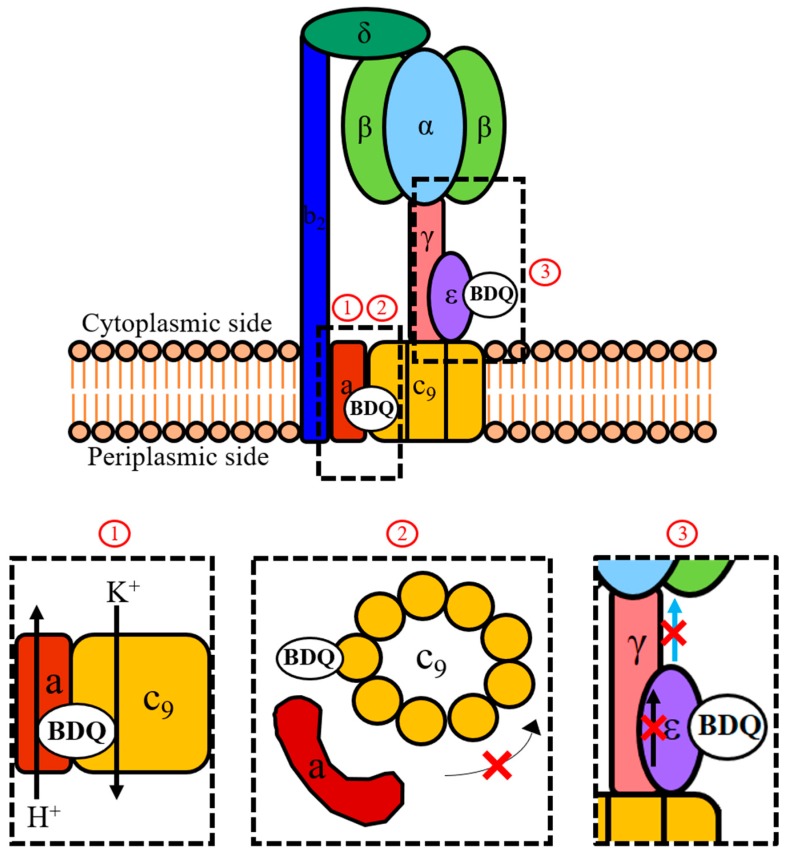
Mechanisms of action of BDQ. Shown is a schematic representation of the mycobacterial F-ATP synthase and the three proposed mechanisms (1, 2, 3) of how BDQ interferes with the activity of this enzyme complex. During ATP synthesis, the proton motive force drives rotation of the *c*-ring (c9) via the flow of protons down the transmembrane pH gradient through the interface between the *a*-subunit and *c*-ring. This rotation is transmitted by the γ- and ε-subunits to the α_3_β_3_-headpiece where it drives ATP synthesis. An indirect mechanism of action of BDQ involves the drug functioning as a H^+^/K^+^ anti-porter from the vicinity of its *c*-ring binding site (1), thereby collapsing the transmembrane pH gradient component of the proton motive force which is required to drive *c*-ring rotation. This causes the uncoupling of electron transport in the electron transport chain (ETC) from ATP synthesis at the F-ATP synthase. The direct mechanisms of action of BDQ involve targeting the *c*-ring (2) and the ε-subunit (3) of the enzyme. Binding to a *c*-subunit stalls rotation of the *c*-ring and thus inhibits ATP synthesis in the catalytic α_3_β_3_-headpiece. Binding to the ε-subunit disrupts its intra-subunit amino acid interaction network. This prevents the transmission of information on *c*-ring rotation throughout the subunit (represented by the black arrow) and to the α_3_β_3_-headpiece through the extended form of its C-terminus (represented by the blue arrow). Comparative analyses of the new BDQ analogue TBAJ-876 with the parental drug showed that new compound retained targeting of both the *c*-ring (2) and ε-subunit (3), suggesting that both mechanisms are required for effective enzyme inhibition. However, TBAJ-876 was uncovered to have lost BDQ’s uncoupler property (1). These findings suggest that targeting both the *c*-ring and ε-subunit of the mycobacterial F-ATP synthase is required for BDQ’s anti-mycobacterial activity, while its uncoupler property is not.

BDQ has been extensively studied and characterized by various groups. These works suggest three distinct mechanisms of action of the drug, all targeting ATP synthesis catalyzed by the mycobacterial F-ATP synthase (Figure 2). The first two mechanisms involve binding to distinct sites on the enzyme itself. Binding to the *c*-subunit causes stalling of rotation of the *c*-ring, while binding to the ε-subunit inhibits its ability to link *c*-ring rotation to ATP synthesis at the enzyme’s α_3_β_3_-headpiece (Figure 2). A third mechanism involves BDQ functioning as an uncoupler of electron transport in the ETC from ATP synthesis at the F-ATP synthase by collapsing the transmembrane pH gradient component of the proton motive force (Figure 2). The large body of data on these mechanisms have led to the proposal that BDQ’s targeting of the *c*-ring and its uncoupler activity are crucial for the drug’s anti-mycobacterial whole cell activity, while the drug’s targeting of the ε-subunit might be negligible. The new BDQ analogue TBAJ-876, which is the structurally (Figure 1) and physico-chemically different from the parental drug, was discovered empirically (without mechanistic guidance) using whole cell activity as readout. This new compound provided the opportunity to revisit BDQ’s mechanisms of action. If all three mechanisms of BDQ are critical for the anti-mycobacterial activity of the drug, the new analogue should have retained all of them. If one of those mechanisms is not critical for the activity of BDQ, it is likely that it would have been lost during the extensive chemical derivatization process. The mechanism of action studies of TBAJ-876 revealed that the compound retained targeting of the *c-*ring. Interestingly, TBAJ-876 also retained targeting of the ε-subunit, suggesting that targeting of both the *c*-ring and ε-subunit is required for effective inhibition of the F-ATP synthase. Surprisingly, TBAJ-876 did not retain BDQ’s uncoupler activity, suggesting that this mechanism is not critical for the anti-mycobacterial activity of this compound class. In conclusion, characterization of the mechanisms of action of TBAJ-876 suggest that dual targeting of both the *c*-ring and the ε-subunit of the mycobacterial F-ATP synthase is required for BDQ’s anti-mycobacterial activity, while its uncoupler property may not be essential for its activity. A detailed understanding of the mechanisms of action of BDQ is of essence if we want to utilize a rational approach for the discovery of a next generation BDQ. The recent TBAJ-876 findings suggest that chemistry programs do not need to focus on retaining BDQ’s uncoupler property in new BDQ analogues to achieve bactericidal activity. However, new analogues may be required to retain targeting of both the *c*-ring and the ε-subunit of the mycobacterial F-ATP synthase to generate effective inhibitors of this enzyme.
